# Comparison of Properties of Stem Cells Isolated from Adipose Tissue and Lipomas in Dogs

**DOI:** 10.1155/2019/1609876

**Published:** 2019-11-20

**Authors:** Takahiro Teshima, Akito Matsuoka, Maika Shiba, Kazuho Dairaku, Hirotaka Matsumoto, Ryohei Suzuki, Hidekazu Koyama

**Affiliations:** Laboratory of Veterinary Internal Medicine, Department of Veterinary Clinical Medicine, School of Veterinary Medicine, Faculty of Veterinary Science, Nippon Veterinary and Life Science University, 1-7-1 Kyonan-cho, Musashino-shi, Tokyo 180-8602, Japan

## Abstract

Adipose-derived mesenchymal stem cells (ADSCs) have been suggested their benefits in regenerative medicine for various diseases. Lipomas, benign neoplasms in adipose tissue, have been reported as a potential source of stem cells. These lipoma-derived mesenchymal stem cells (LDSCs) may be useful for regenerative medicine. However, the detailed characteristics of LDSCs have not been fully elucidated. This study investigated the cellular proteomics and secretomes of canine LDSCs in addition to morphology and proliferation and differentiation capacities. Some LDSCs isolated from canine subcutaneous lipomas were morphologically different from ADSCs and showed a rounded shape instead of fibroblast-like morphology. The phenotype of cell surface markers in LDSCs was similar to those in ADSCs, but CD29 and CD90 stem cell markers were more highly expressed compared with those of ADSCs. LDSCs had noticeably high proliferation ability, but no significant differences were observed compared with ADSCs. In regard to differentiation capacity compared to ADSCs, LDSCs showed higher adipogenesis, but no differences were observed with osteogenesis. Cellular proteomic analysis using two-dimensional gel electrophoresis revealed that over 95% of protein spots showed similar expression levels between LDSCs and ADSCs. Secretome analysis was performed using iTRAQ and quantitative cytokine arrays. Over 1900 proteins were detected in conditioned medium (CM) of LDSCs and ADSCs, and 94.0% of detected proteins showed similar expression levels between CM of both cell types. Results from cytokine arrays including 20 cytokines showed no significant differences between CM of LDSCs and that of ADSCs. Our results indicate that canine LDSCs had variability in characteristics among individuals in contrast with those of ADSCs. Cellular proteomics and secretomes were similar in both LDSCs and ADSCs. These findings suggest that LDSCs may be suitable for application in regenerative medicine.

## 1. Introduction

Mesenchymal stem cells (MSCs) are adult multipotential progenitors with demonstrated important utility in regenerative medicine. Adipose tissue-derived MSCs (ADSCs) are stem cells derived from adipose tissue and have several advantages, as adipose tissues are abundant and are easily accessible to obtain cells [1]. Therefore, several studies have demonstrated the usefulness of ADSCs in tissue engineering and regenerative medicine for various diseases [2, 3].

Lipomas are common soft tissue mesenchymal neoplasms that can be located in any part of the body. Lipoma-derived MSCs (LDSCs) were first reported in 2007 [4] and show higher proliferation compared with ADSCs. Several studies reported the properties of LDSCs [5–10], and most reports investigated cell surface markers, proliferation, and multilineage differentiation including adipogenesis, osteogenesis, and chondrogenesis, with only functional research on anti-inflammatory effects [9]. These studies suggested that LDSCs were a good source of MSCs and might be as useful as ADSCs. One advantage of ADSCs is that adipose tissues can be obtained with minimal invasive procedures such as liposuction aspirates or adipose tissue biopsies, but it is necessary for invasion even though minimal. In contrast, to obtain lipoma tissues is a process of surgery treatment not a target. Notably, the use of lipomas obtained after surgery could be a very attractive source of regenerative medicine.

The aim of this study was to evaluate the properties of LDSCs compared with ADSCs using cellular proteomic and secretome analyses and explore the possibility of their use in regenerative medicine.

## 2. Materials and Methods

### 2.1. Tissue Samples

Lipoma tissue samples were obtained from five dogs at the Veterinary Medical Teaching Hospital of Nippon Veterinary and Life Science University. The solitary subcutaneous masses were surgically resected under general anaesthesia. Histologically, all masses were composed of proliferation of mature fat cells having only a slight variation in cellular size and shape without cellular atypia, containing collagen, or clusters of small blood vessels. Therefore, all masses were diagnosed as lipoma. Normal adipose tissue samples were aseptically collected from falciform ligament fat of four healthy beagles under general anaesthesia. Detailed information on the dogs is listed in Table 1. Dogs were handled in accordance with the animal care guidelines of the Institute of Laboratory Animal Resources, Nippon Veterinary and Life Science University, Japan. The Institutional Animal Care and Use Committee of Nippon Veterinary and Life Science University approved the experimental design.

### 2.2. Isolation and Culture of Canine ADSCs and LDSCs

Adipose tissues and lipomas were washed extensively in PBS, minced, and digested with collagenase type I (Sigma-Aldrich) at 37°C for 45 min with intermittent shaking. After washing with PBS and centrifuging, the pellets containing the stromal vascular fraction were resuspended, filtered through a 100 *μ*m nylon mesh, and incubated overnight in high-glucose Dulbecco's modified Eagle's medium (H-DMEM) supplemented with 10% fetal bovine serum (FBS; Nichirei Bioscience) and a 1% antibiotic-antimycotic solution (Thermo Fisher Scientific) in a humidified atmosphere with 5% CO_2_ at 37°C. Unattached cells were removed by changing the medium, and the attached cells were washed twice with PBS. Thereafter, the medium was replaced every 3–4 days. Once cells reached 80%–90% confluence, the cells were detached with trypsin-EDTA solution (Sigma-Aldrich) and passaged repeatedly.

### 2.3. Phenotype of ADSCs and LDSCs

ADSCs and LDSCs at passages 2, 4, and 6 were analyzed by flow cytometry. The cells were placed in fluorescence-activated cell sorting (FACS) tubes (BD Biosciences; 2 × 10^5^ cells/tube), washed with FACS buffer (PBS containing 2% FBS), and then incubated with the following fluorescein (FITC)- or phycoerythrin (PE)-conjugated antibodies: anti-CD14-FITC (clone M5E2; BD Pharmingen), anti-CD29-PE (clone TS2/16; BioLegend), anti-CD34-PE (clone 1H6; R&D Systems), anti-CD44-PE (clone IM7; BioLegend), anti-CD45-FITC (clone YKIX716.13; eBioscience), and anti-CD90-PE (clone YKIX337.217; eBioscience) or their respective isotype controls [11, 12]. The cells were washed twice with FACS buffer and resuspended in 500 *μ*l FACS buffer. Fluorescence was evaluated by flow cytometry using a FACSCalibur instrument (BD Biosciences). Data were analyzed using WinMDI 2.9 analysis software.

### 2.4. Differentiation Assays

For osteogenic differentiation, ADSCs and LDSCs at passages 2, 4, and 6 were seeded in 6-well plates (5.0 × 10^3^ cells/cm^2^) and incubated in H-DMEM supplemented with 10% FBS and 1% antibiotic-antimycotic solution for 24 h. The medium was then changed to osteogenic medium (Cell Applications) [13]. The medium was changed twice weekly. For osteogenic analysis, mineral deposits were quantitatively analyzed by von Kossa staining after 21 days.

For adipogenic differentiation, ADSCs and LDSCs at passages 2, 4, and 6 were seeded in 6-well plates (8 × 10^3^ cells/cm^2^) and cultured in H-DMEM supplemented with 10% FBS and 1% antibiotic-antimycotic solution until confluency. The medium was changed to canine adipocyte differentiation medium (Cell Applications) [13]. The medium was changed twice weekly. Adipogenesis was analyzed by Oil Red O staining after 21 days.

Positive-stained areas were measured with ImageJ. Four fields were randomly selected from culture dishes divided equally into four regions.

### 2.5. Proliferation Assay

Cell proliferation was determined using an MTT assay kit (Roche Diagnostics). ADSCs and LDSCs at passage 2 to 6 were seeded in 96-well flat-bottomed plates (3 × 10^3^ cells/well). MTT assays were performed every 24 h for 3 days according to the manufacturer's instructions.

### 2.6. Senescence-Associated *β*-Galactosidase Assay

ADSCs and LDSCs at passage 3 were seeded in a 6-well plate (5 × 10^3^ cells/cm^2^) in duplicate. After 24 h, *β*-galactosidase expression was detected using a Senescence *β*-Galactosidase Staining Kit (Cell Signaling Technology) according to the manufacturer's instructions. The number of positive (blue) and negative (not colored) cells was counted in each sample in at least five random fields under a light microscope.

### 2.7. Two-Dimensional Gel Electrophoresis (2-DE)

Cells at passages 2 and 5 at 70%–80% confluence were washed three times using ice-cold PBS and resuspended in lysis buffer (7 M urea, 2 M thiourea, 2% CHAPS, 20 mM DTT, and 1% protease inhibitor cocktail). After adding 1 ml of lysis buffer, cells were immediately scraped using a cell scraper and collected into 1.5 ml microtubes. Samples were incubated at room temperature for 15 min and vortexed occasionally. For alkylation, 5 *μ*l of 99% N,N-dimethylacrylamide was added and samples were incubated at room temperature for 30 min on a rotary shaker. After adding 10 *μ*l of 2 M DTT, samples were centrifuged at 4°C and 12500 g for 30 min. Supernatants were collected into microtubes and stored at -80°C. Protein concentrations were measured using the BCA Protein assay kit. For isoelectric focusing (IEF), ZOOM IPG Strip (Thermo Fisher Scientific) with a 3–10 nonlinear pH range was reswelled for 1 h with 150 *μ*l of IEF buffer (7 M urea, 2 M thiourea, 2% CHAPS, 20 mM DTT, 0.5% ZOOM Carrier Ampholytes 3–10 (Thermo Fisher Scientific), and 0.002% bromophenol blue) containing 50 *μ*g protein. IEF was performed using a ZOOM IPG Runner instrument (Thermo Fisher Scientific) at 175 V for 20 min, after which the voltage was increased 175 V to 2000 V over 45 min then held for 30 min. After IEF, strips were equilibrated for 15 min using 5 ml of equilibration buffer containing 1.25 ml of NuPAGE LDS sample buffer (Thermo Fisher Scientific) and 0.5 ml of Sample Reducing Agent (Thermo Fisher Scientific). Samples were then separated on 4–12% Bis-Tris ZOOM Gels (Thermo Fisher Scientific). Samples were run in triplicate. After labeling with fluorescent dye Flamingo (Bio-Rad), gels were scanned on a Molecular Imager FX Pro (Bio-Rad). Preparative gels were stained by Silver Stain for Mass Spectrometry (Thermo Fisher Scientific).

Images were analyzed with PDQuest 2-D Analysis Software Ver 7.3 (Bio-Rad). Protein spots were identified using the automatic spot detection algorithm. Individual spot volumes were normalized against total spot volumes.

### 2.8. Protein Identification by MALDI-TOF

To identify differentially expressed proteins (spot Nos. 1, 6, and 11) by peptide mass fingerprinting, protein spots were excised from the preparative gels, digested with trypsin (Promega), mixed with *α*-cyano-4-hydroxycinnamic acid in 50% acetonitrile and 0.1% TFA, and subjected to MALDI-TOF analysis (Microflex LRF 20, Bruker Daltonics) as described [14]. Spectra were collected from 300 shots per spectrum over *m*/*z* range 600–3000 and calibrated by two-point internal calibration using trypsin autodigestion peaks (*m*/*z* 842.5099, 2211.1046). The peak list was generated using Flex Analysis 3.0. The threshold for peak-picking was as follows: 500 for minimum resolution of monoisotopic mass and 5 for *S*/*N*. The search program MASCOT, developed by Matrix Science (http://www.matrixscience.com/), was used for protein identification by peptide mass fingerprinting. The following parameters were used for the database search: trypsin as the cleaving enzyme, a maximum of one missed cleavage, iodoacetamide as a complete modification, oxidation (Met) as a partial modification, monoisotopic masses, and a mass tolerance of ±0.1 Da. PMF acceptance criteria are probability scoring.

### 2.9. ADSC- and LDSC-Conditioned Medium

To prepare the ADSC- and LDSC-conditioned medium (ADSC-CM and LDSC-CM), ADSCs and LDSCs at passage 2 were separately seeded (2.5 × 10^4^ cells/cm^2^) in H-DMEM supplemented with 10% FBS and 1% antibiotic-antimycotic solution and incubated overnight. Adherent cells were washed and further incubated in FBS-free H-DMEM for 36 h. The medium was collected, filtered through a 0.45 *μ*m filter, and then stored at -80°C until analysis.

### 2.10. iTRAQ Proteomic Analysis of ADSC- and LDSC-CM

iTRAQ proteomic analysis was performed with liquid chromatography tandem mass spectrometry (LC-MS/MS) analysis. Protein concentrations of ADSC-CM and LDSC-CM were determined using the BCA protein assay kit (Thermo Fisher Scientific). The medium was concentrated by ultrafiltration (Agilent Technologies) and adjusted to a concentration of 5 *μ*g/*μ*l using dissolution buffer. Each sample was digested with 1 *μ*g/*μ*l trypsin solution (SCIEX) at 37°C for 24 h and then desalted using a Sep-Pak Light C18 cartridge (Waters Corporation). Peptide samples from each medium were labelled using the iTRAQ reagent-multiplex assay kit (SCIEX) as follows: ADSC-CM from case A with 113 tag, ADSC-CM from case B with 114 tag, LDSC-CM from case H with 115 tag, and LDSC-CM from case E with 116 tag. All samples were mixed, fractionated using strong cation exchange chromatography with a Cation Exchange Buffer Pack (SCIEX), and eluted at various concentrations (25, 50, 75, 100, 150, and 350 mM). Eluted samples were desalted using a Sep-Pak Light C18 Cartridge. After washing with buffer (0.1% formic acid (FA)), peptides were eluted with elution buffer (70% acetonitrile (ACN), 0.1% FA). Each eluted sample was dried and resuspended in 30 *μ*l of buffer (5% ACN, 0.1% FA).

The analysis was performed using a 5600 TripleTOF (SCIEX) interfaced with a DiNa LC system (KYA Technologies) at a flow rate of 150 nl/min. Relative abundance quantitation and peptide and protein identification were performed using ProteinPilot 4.5 beta (SCIEX). MS and MS/MS data were searched for homologs in *Canis lupus familiaris* using the UniProtKB (http://www.uniprot.org). The false discovery rate (FDR) was calculated, and high-confidence protein identifications were obtained using a Global FDR from Fit 1.0% at the peptide level. Quantitative estimates provided for each protein by ProteinPilot were used: the fold change ratios of differential expression between labeled protein extracts and the *P* value representing the probability that the observed ratio is different from 1 by chance. We selected 0.5-fold change as a cutoff to classify downregulated proteins and 1.5-fold change as a cutoff to classify upregulated proteins. Gene Ontology (GO) term enrichment analysis was performed for all identified proteins using the MicroArray Data Analysis Tool Ver3.2 (Filgen).

### 2.11. Cytokine Array of ADSC- and LDSC-CM

The concentrations of secreted cytokines from conditioned medium from ADSCs and LDSCs (from cases E, G, H, and I) were quantified via a Quantibody Canine Cytokine Arrays 1 and 2 (RayBiotech) including IL-1*β*, IL-2, IL-6, IL-8, IL-10, IL-12p40, IL-17A, GM-CSF, MCP-1, RAGE, SCF, TNF*α*, VEGF-A, EPO, FGF-7, HGF, HGF R, IFN*γ*, MIP-1*β*, and TNF RI. The array was performed according to the manufacturer's instructions, and the resulting glass slide was scanned using a GenePix 4400A microarray scanner (Molecular Devices). Collected images were quantified using an Array-Pro Analyzer Ver 4.5 (Media Cybernetics).

### 2.12. Statistical Analysis

The normality of the data was first assessed using the Chi-squared test for goodness of fit. The normally distributed data are presented as the mean ± standard deviation, and the nonnormally distributed data are presented as the median and range. Differences between two groups were analyzed with the Student's *t*-test or Mann-Whitney's *U* test. The MTT assay results were analyzed by two-way ANOVA followed by Tukey-Kramer's post hoc tests. A value of *P* < 0.05 was considered as statistically significant. Statistical analyses were performed using Excel 2010 with add-in software Statcel 3 except for data on protein spot from two-dimensional gel electrophoresis (2-DE). Regarding the data of protein spot from two-dimensional gel electrophoresis, statistical analyses were performed using PDQuest 2-D Analysis Software Ver 7.3.

## 3. Results

### 3.1. Morphology of Stromal Cells

**Figure 1:**
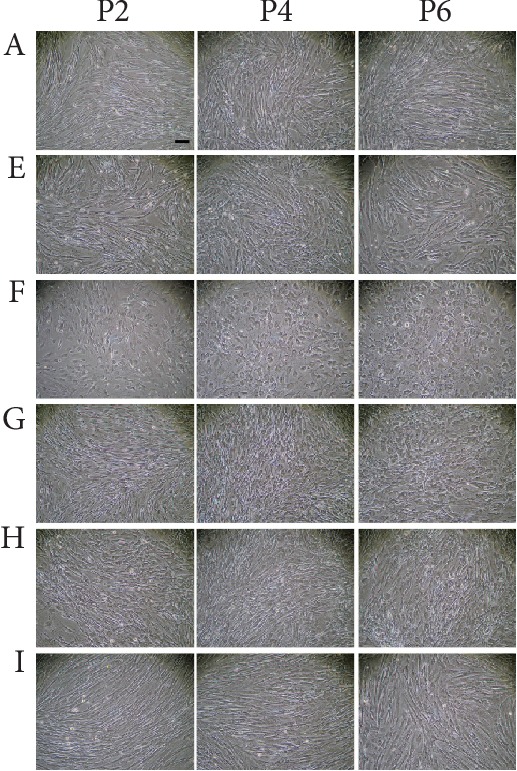
Morphology of lipoma-derived mesenchymal stem cells and adipose tissue-derived mesenchymal stem cells. Adipose tissue-derived mesenchymal stem cells (ADSCs) isolated from case A and lipoma-derived mesenchymal stem cells (LDSCs) isolated from cases E, F, G, H, and I are shown. ADSCs show a fibroblast-like shape. LDSCs from cases E, H, and I are similar in shape of ADSCs, but those from cases F and G are not like ADSCs and instead show a rounded shape. Bar = 100 *μ*m.

Both ADSCs and LDSCs were successfully cultured and expanded as described in Materials and Methods. The morphology of ADSCs was very similar and typical fibroblast-like, with no significant differences observed between individuals as well as after cell passage. Most LDSCs were also similar in shape to ADSCs, but cells in some individuals (case F at all passages and case G at passage 6) showed a rounded shape unlike ADSCs (Figure 1).

### 3.2. Cell Surface Markers

The majority of both ADSCs and LDSCs expressed the established MSC markers CD29, CD44, and CD90, and very few expressed CD14, CD34, or CD45 (Table 2). A higher expression of CD29 was noticed in LDSCs at passages 5 and 6 (*P* < 0.05) compared with that of ADSCs. The expression of CD90 was significantly higher in LDSCs at passages 3, 4, and 6 than that of ADSCs (*P* < 0.05).

### 3.3. Adipogenic and Osteogenic Differentiation

**Figure 2:**
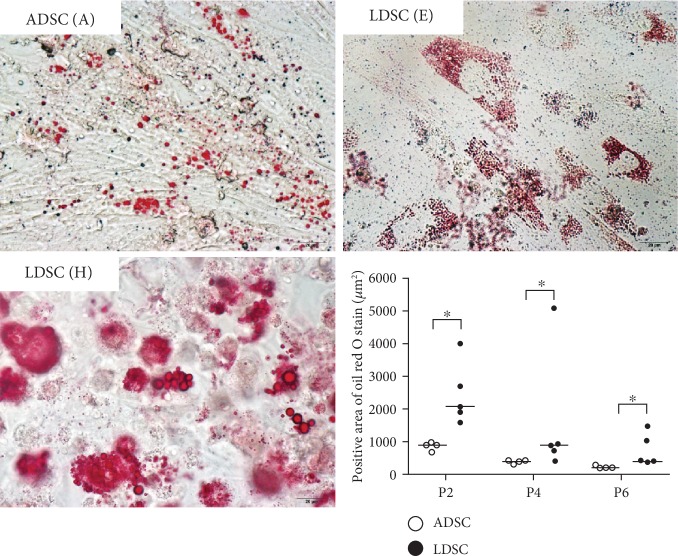
Adipogenic differentiation of canine ADSCs and LDSCs. Adipogenic differentiation was identified by Oil Red O staining. LDSCs isolated from case H showed multiple large lipid droplets. Positive area of Oil Red O stain was significantly higher in LDSCs compared with that in ADSCs at P2, P4, and P6. ^∗^*P* < 0.05 vs. ADSC.

**Figure 3:**
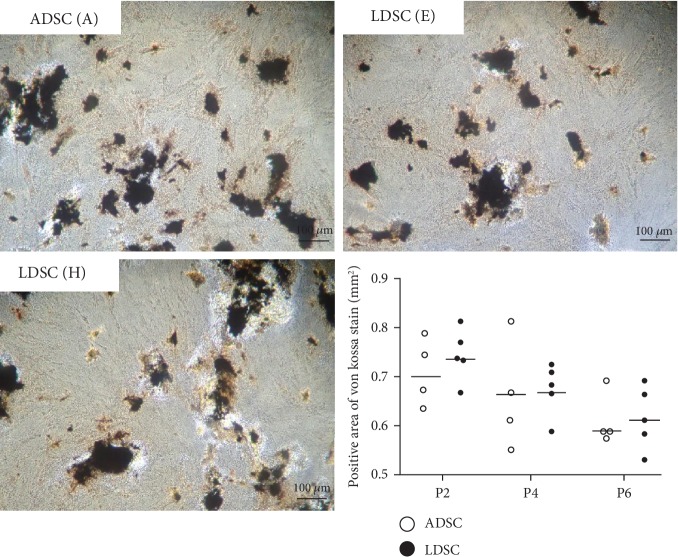
Osteogenic differentiation of canine ADSCs and LDSCs. Osteogenic differentiation was identified by von Kossa staining. There were no significant differences in positive von Kossa staining areas between ADSCs and LDSCs at P2, P4, and P6.

Both ADSCs and LDSCs in various passages were successfully induced to adipogenesis and osteogenesis. After 21 days of adipogenic differentiation, more lipid droplets were confirmed in LDSCs compared with ADSCs (Figure 2). LDSCs from one case (H) showed multiple and large lipid droplets through passages. Osteogenic differentiation of all of LDSCs was similar to that of ADSCs, and there are no significant differences in the von Kossa-stained area (Figure 3).

### 3.4. Cell Proliferation Capacity

**Figure 4:**
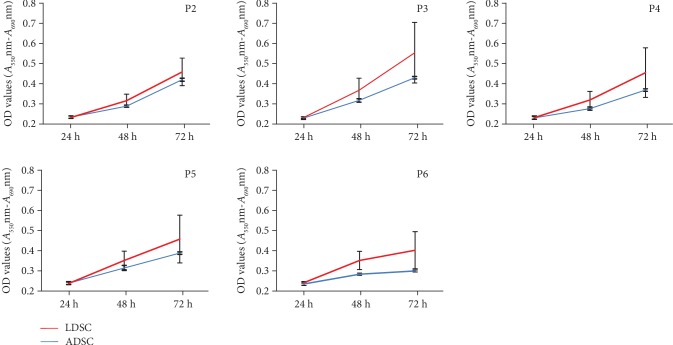
MTT assays through passages. The proliferation rates of LDSCs were slightly higher but not significantly different from those of ADSCs. Data are expressed as the mean ± SD.

**Figure 5:**
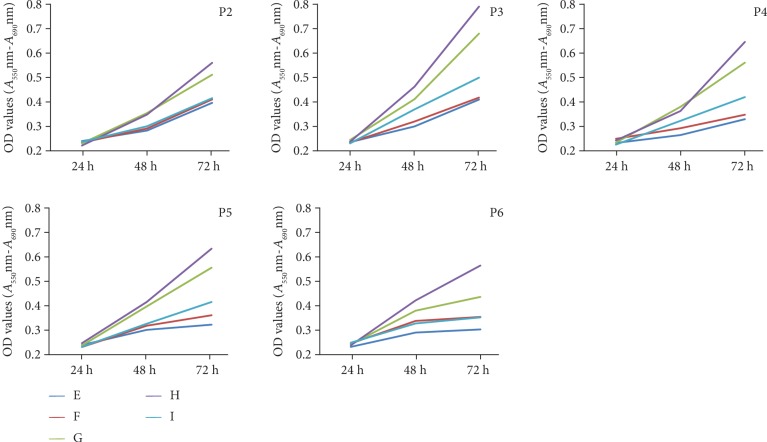
MTT assays of LDSCs at various passages. LDSCs isolated from cases G and H showed relatively higher OD values compared with other cases in various passages.

To determine the proliferation potential at various passages, MTT assays were performed. As shown in Figure 4, there were no significant differences in OD values in all passages between ADSCs and LDSCs, but the proliferation ability of LDSCs was higher than that of ADSCs. The highest proliferation ability was observed in both ADSCs and LDSCs at passage 3 (OD values at 72 h; ADSCs were 0.44 ± 0.01, LDSCs were 0.56 ± 0.15). The proliferation ability in ADSCs was similar among individuals, but LDSCs showed different proliferations among individuals. Some LDSCs (cases G and H) showed a high proliferation ability through passages (Figure 5).

### 3.5. Senescence-Associated *β*-Galactosidase Expression

**Figure 6:**
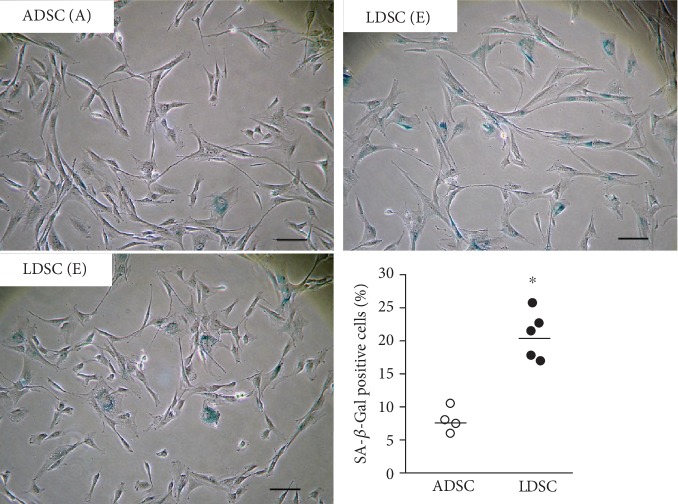
SA-*β*-gal expression of ADSCs and LDSCs at P3. The percentage of SA-*β*-gal-positive cells was significantly higher in LDSCs compared with that in ADSCs. ^∗^*P* < 0.05 vs. ADSC.

As an indicator for cellular senescence, the expression of senescence-associated *β*-galactosidase (SA-*β*-gal) was determined by a histochemical staining method. Both ADSCs and LDSCs at P3 observing the highest proliferation ability using the MTT assay were analyzed. As shown in Figure 6, the percentage of SA-*β*-gal-positive cells was significantly higher in LDSCs (20.8 ± 3.3%) than that of ADSCs (8.0 ± 1.6%) (*P* < 0.05).

### 3.6. Cellular Proteomics

**Figure 7:**
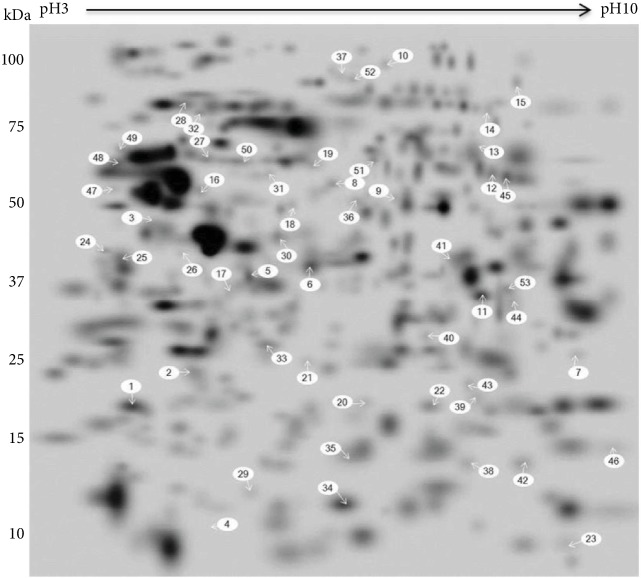
2-DE master gel of ADSCs and LDSCs. Canine ADSCs and LDSCs at P2 and P5 were separated using 2-DE electrophoresis in a dry strip pH 3–10 for the first dimension and a 4–12% SDS-PAGE for the second dimension and stained with fluorescent dye. The spots that showed significant differences are indicated with numbers.

Canine ADSCs and LDSCs at P2 and P5 were separated using 2-DE electrophoresis. Triplicates gels from each sample showed high reproducibility when run under identical conditions. Each spot in the gel was assigned a unique arbitrary number during the matching process. A representative 2-DE gel is shown in Figure 7. An average of 443 ± 86 protein spots reflected the whole-cell proteome of ADSCs and LDSCs. Matching protein spots (307–340 spots) were compared between ADSCs and LDSCs at P2 and P5 using PDQuest software. At P2, 95.3% (324/340) of spots showed no significant differences in levels, while 13 spots in ADSCs and 3 spots in LDSCs showed significantly higher expression levels compared with the respective group (Table 3). At P5, 97.4% (300/309) of spots showed no significant differences in levels, but 5 spots in ADSCs and 4 spots in LDSCs showed significantly higher expression levels. In the comparison between P2 and P5, 89.9% (276/307) and 96.7% (323/334) of spots showed no significant differences in ADSCs and LDSCs, respectively.

### 3.7. Protein Identification

**Figure 8:**
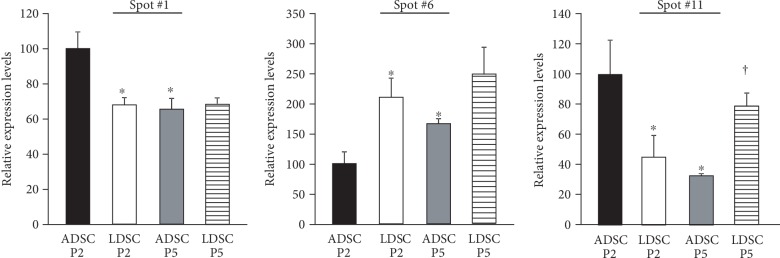
Comparison of relative protein expression levels identified by MALDI-TOF analysis. ^∗^*P* < 0.05 vs. ADSC P2; ^†^*P* < 0.05 vs. LDSC P2.

Three spots (Nos. 1, 6, and 11), which were clear expressions, able to be excised manually, and significant differences in ADSCs and LDSCs or P2 and P5 were identified by MALDI-TOF analysis. The relative expression levels of the three spots are shown in Figure 8. The spot No. 1, which showed a higher expression level in ADSCs than LDSCs at P2 and higher levels in ADSCs at P2 than P5, was identified as translationally controlled tumor protein (TCTP) isoform X2. Spot No. 6, which showed higher expression levels in LDSCs compared with ADSCs at P2 and higher levels in ADSCs at P5 than P2, was identified as annexin A1 (ANXA1). Spot No. 11, which showed a lower expression level in LDSCs than ADSCs at P2, lower level in ADSCs at P5 than P2, and lower levels in LDSCs at P2 than P5, was identified as pirin isoform X2.

### 3.8. Secreted Proteins in Conditioned Medium

To identify soluble factors from ADSCs and LDSCs, iTRAQ proteomic analysis was performed using ADSC- and LDSC-CM. The morphology, differentiation potential, and proliferation capacity were similar among ADSCs, but differences were observed among LDSCs. Therefore, LDSC-CM from cases H and E, which showed higher and lower proliferation abilities in MTT assays, respectively, were analyzed. iTRAQ proteomic analysis revealed 1910 proteins except for those identified by de novo sequences (Supplemental [Supplementary-material supplementary-material-1]). Using 0.5-fold change as a cutoff to classify downregulated proteins and 1.5-fold change as a cutoff to classify upregulated proteins, 94.0% (1795/1910) of detected proteins showed similar expression levels between ADSC- and LDSC-CM. Upregulated and downregulated proteins in ADSC- and LDSC-CM are shown in Table 4. According to Gene Ontology analysis, all identified 1910 proteins were categorized as follows: 72.9% in biological process, 10.0% in cellular component, and 17.1% in molecular function and related to various functions (Supplemental [Supplementary-material supplementary-material-1]).

### 3.9. Secreted Cytokines in Conditioned Medium

**Figure 9:**
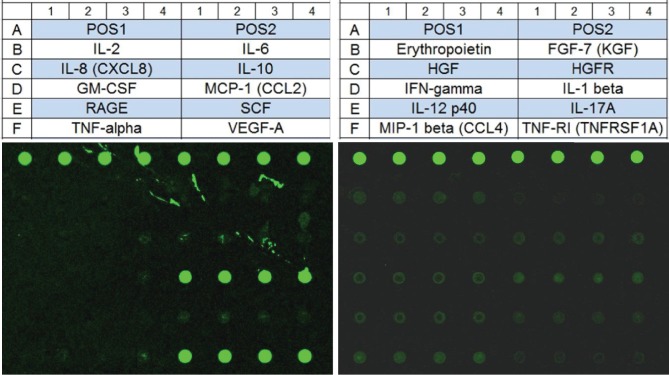
Measurement of secreted cytokines in conditioned medium of ADSCs and LDSCs. Twenty cytokines were examined by quantitative cytokine arrays. The bottom two scanning images are glass slides from conditioned medium of ADSC isolated from case A.

Data from two arrays demonstrated that ADSCs and LDSCs secrete cytokines (Figure 9). All data of cytokine arrays are shown in Table 5. GM-CSF was not detected in any sample. Concentrations of eleven cytokines including IL-1*β*, IL-2, IL-6, IL-8, IL-10, IL-12p40, IL-17A, MCP-1, RAGE, SCF, TNF*α*, VEGF-A, EPO, FGF-7, HGF, HGF R, IFN*γ*, MIP-1*β*, and TNF RI did not show statistically significant differences between ADSC- and LDSC-CM.

## 4. Discussion

MSCs can be isolated from bone marrow, adipose tissue, umbilical cord, dental pulp, and amniotic fluid [15, 16]. ADSCs can be obtained by less invasive manners than bone marrow-derived MSCs (BMSCs) and with easily abundant number of cells than others. These advantages have led to many reports showing the efficacy of ADSCs in various diseases, including veterinary medicine [17, 18].

Despite the close histological similarity to normal adipose tissue, cytogenetic investigations revealed that human lipomas show a high incidence of chromosomal aberrations, such as translocations involving 12q13-15, locus interstitial deletions of 13q, and rearrangements involving 8q11-13 locus [19]. There is only one report of cytogenetic investigations on canine lipomas [20]. This study showed that clonal aberrations were observed in seven cases such as trisomy 27, trisomy 13, and derivative chromosomes X, 2, and 7. There are genetic differences between adipose tissue and lipoma, but several studies reported that MSCs could be isolated from lipomas and might be used for regenerative medicine as in ADSCs [5–10]. To evaluate the possibility of using LDSCs as stem cell therapy in the clinic in this study, we compared the properties of LDSCs with ADSCs in terms of not only morphology, cell surface markers, and differentiation and proliferation abilities but also intracellular protein expression, secreted proteins, and cytokines.

Morphologically, all ADSCs from falciform ligament fat of healthy beagles were fibroblast-like across various passages. However, some LDSCs were different from ADSCs and showed rounded shape. Notably, other studies showed that the morphology of LDSCs is similar to ADSCs and there are no morphological differences between ADSCs and LDSCs across passages [4, 8, 10]. The sources of LDSCs in this study were all subcutaneous lipomas, which are benign tumors, and did not include fibrolipomas, angiolipomas, or liposarcomas. LDSCs isolated from case F were shown most specific shape of cells, but there were no noticeable differences in terms of characteristics of case F compared with other cases besides body size.

Cell surface markers, such as CD29, CD44, and CD90, have been used to characterize canine ADSCs. Other markers, such as hematopoietic markers CD34 and CD45, need to be absent or show very low expression. In this study, both ADSCs and LDSCs across passages showed the phenotypic expression pattern of canine ADSCs. The expressions of cell surface markers of LDSCs isolated from human lipomas showed no significant differences compared with ADSCs, but a slightly higher expression of CD44 was observed in ADSCs compared with LDSCs [9, 10]. In our study, CD44 expression in LDSCs was similar to ADSCs, but CD29 and CD90 showed higher expression in LDSCs compared with ADSCs. CD29, also known as integrin *β*1, is a cell surface adhesion receptor that mediates the cell-extracellular matrix and cell-cell interactions. CD90, also known as Thy-1, has been implicated in MSC self-renewal and differentiation [21], but its function in MSC biology remains unclear. Some studies have reported the correlation of CD29 and CD90 with MSC biology [22–24]. A study using murine ADSCs examined reprograming efficiency by transduction with four standard factors (Oct4, Sox2, Klf4, and c-Myc) and reported that CD90^Hi^ cells had greater reprogramming capacity than CD90^low^ cells [22]. In regard to differentiation potential, double positive cells for CD29 and CD90 sorted from rat BMSCs and ADSCs demonstrated a reduced adipogenic and osteogenic differentiation capacity compared with unsorted cells [24]. Therefore, the authors concluded that maintaining heterogeneity within MSC cultures may be of benefit for improved differentiation. In addition, a study analyzing the effect of CD90 knockdown on proliferation, morphology, and differentiation of human MSCs isolated from dental pulp, adipose tissue, and amniotic fluid showed that reduced CD90 enhanced the osteogenic and adipogenic differentiation of MSCs but did not affect morphology and proliferation [23]. This study suggested that CD90 controls the differentiation of MSCs by acting as an obstacle in the pathway of differentiation commitment. In our study, higher CD29 and CD90 expressions were observed in LDSCs. However, adipogenic differentiation capacity assessed by the positive Oil Red O staining area was higher in LDSCs than that of ADSCs, but osteogenic differentiation ability showed similar results. Previous studies showed that the adipogenic differentiation capacity in LDSCs was lower or similar to ADSCs, and osteogenic differentiation capacity in LDSCs was similar to ADSCs [4, 10]. LDSC isolated from case H with fibroblast-like-shaped cells showed abundant lipid droplets after adipogenic differentiation, but similar osteogenic differentiation capacity compared with ADSCs. Our results of phenotype cell surface markers and differentiation potential in canine LDSCs were not concordant with the previous findings regarding CD29 and CD90 expression levels and differentiation capacity in ADSCs, suggesting that the differentiation ability in LDSCs may vary among individuals or species.

The MTT assay showed that the proliferation ability of LDSCs was higher than ADSCs, but there were no significant differences from P2 to P6. The proliferation ability of ADSCs was similar among four samples across passages while that of LDSCs showed variation among individuals. LDSCs isolated from cases G and H showed relatively higher proliferation ability than the other three cases. A previous study on population doubling levels reported no differences in expansion capacity between ADSCs and LDSCs [8]. In contrast, another study using cumulative population doubling level (cpdl) reported that the cpdl of LDSCs was higher than that of ADSCs [4]. In canine ADSCs, there was an age-dependent change in proliferation ability. The previous study documented the comparison of the cpdl of canine ADSCs isolated from 7-month-old dogs and 10- to 11-year-old dogs, and the result in the cpdl shows significantly higher ADSCs from young donors than those of old donors in dogs [25]. The donors of LDSCs in our study were significantly older than those of ADSCs (14-month-old donors for ADSCs, 126-month-old donors for LDSCs). The expression of SA-*β*-gal as a cellular senescence marker was significantly higher in LDSCs than that of ADSCs. Therefore, our results suggested that the proliferation ability of LDSCs was similar to or higher than that of ADSCs, and the proliferation ability in LDSCs was donor-dependent rather than age-dependent.

In this study, we compared the cellular proteomes between ADSCs and LDSCs using 2-DE gel analysis. In the comparison of protein expression patterns between ADSCs and LDSCs, no single characteristic molecule in either cell type could be identified. In the comparison of P2 and P5, protein spots with higher expression levels were observed more frequently in ADSCs than LDSCs. A proteomic analysis of MSCs isolated from different tissues has been reported in humans [26], and a study comparing MSCs among isolated tissues demonstrated that ADSCs were very similar to BMSCs. No research has compared passages in ADSCs. However, the comparison between P3 and P7 BMSCs showed that proteins of the functional categories “structural components and cellular cytoskeleton” and “folding and stress response proteins” are less abundant in P7 cells compared with P3 cells, while proteins in “energy metabolism,” “cell cycle regulation and aging,” and “apoptosis” are more abundant [27]. In our study, we compared the relative protein expression levels between ADSCs and LDSCs at P2 and P5, and only three spots showing significantly different expression levels were identified by PMF. Spot number 1, which showed higher expression levels in P2 of ADSCs than LDSCs and P5 of ADSCs, was identified as TCTP. TCTP is a highly conserved protein present in all eukaryotic organisms and a multifunctional protein that is highly regulated during adaptation of cells to alterations in physiological conditions, such as growth induction and tumorigenesis, and many molecular, cellular, and indeed extracellular functions have been ascribed [28, 29]. TCTP1 contributes to the adipocyte lineage commitment from C3H10T1/2 pluripotent stem cells, but its function in MSCs is not clear [30]. Spot number 6, which showed higher expression levels in P2 of LDSCs compared with P2 of ADSCs and in P5 of ADSCs compared with P2 of ADSCs, was identified as ANXA1. ANXA1, also known as lipocortin 1, is a member of the superfamily of Ca^2+^ and phospholipid binding proteins, with widespread tissue localization [31]. High expression levels of ANXA1 in cells of the hematopoietic lineage are consistent with its well-documented anti-inflammatory properties [32]. ANXA1 both suppresses phospholipase A2, thereby blocking eicosanoid production, and inhibits various leukocyte inflammatory events. Proteomic analysis of BMSCs using 2-DE showed that the annexin family is categorized into the apoptosis function group, and ANXA1 showed a higher expression in P7 of BMSCs than P3 of BMSCs [27]. The result of older passage cells having abundant annexin A1 was similar to our results of ADSCs, but the relation of expression levels and anti-inflammatory function was not documented. Spot number 11, which was expressed lower in P2 of LDSCs compared with P2 of ADSCs and expressed lower in P5 of ADSCs compared with P2 of ADSCs, while expressed higher in P5 of LDSCs than P2 of LDSCs, was identified as pirin isoform X2. Pirin is highly expressed in mammals, plant fungi, and prokaryotes [33]. While the function of pirin is poorly understood, pirin homologs are known to regulate many biological processes. The functions of pirin in MSCs are also unknown, but cells expressing pirin show a spindle-like morphology [34].

The therapeutic effects of transplanted ADSCs were initially thought to be mediated by the migration of ADSCs to the sites of damaged tissue and differentiation into specialized cells [35]. However, only a small proportion of cells have been observed to truly engraft in damaged host tissue. In our previous study, the curative effects of ADSCs on the acute hepatic injury model in dogs were observed, but the number of ADSCs injected via the peripheral vein engrafted in the liver was not so high [11]. Recently, transplanted MSCs do not necessarily have to be engrafted to damaged tissue, and it is proposed that MSCs might exert their therapeutic effects through secreted factors [36, 37]. Such soluble factors may provide a supportive microenvironment in the damaged tissue for cell survival, cell renewal, and differentiation, modulate inflammatory reaction, and induce angiogenesis [36, 37]. The ADSC secretome is a rich source of proteins including cytokines, chemokines, and growth factors [38, 39]. Numerous studies have shown the beneficial effects that the ADSC secretome exerts in angiogenesis, immunomodulation, wound healing, and tissue regeneration, among other effects. In this study, we performed proteomic profiling of LDSC- and ADSC-CM by iTRAQ and quantitative antibody array. Over 1900 proteins were identified, and almost all proteins secreted from LDSCs showed similar expression levels to ADSCs, and the number of highly secreted proteins from LDSCs was more than that from ADSCs. Gene Ontology revealed that the soluble factors from LDSCs and ADSCs were related to numerous functions. Only one functional study of LDSCs has been reported, which suggested that LDSC-CM has slightly more pronounced effects on the activation of macrophages and showed that LDSCs stimulated wound healing in a similar manner of ADSCs [9]. Our results suggest that LDSC-CM may show the same clinical benefits as ADSC-CM.

Cellular heterogeneity exists even within seemingly homogenous stem cell populations, which are influenced by extrinsic microenvironmental factors or intrinsic factors. Abundant evidence has demonstrated that MSCs in culture are intrinsically heterogeneous in phenotypes and functions [40–44]. Even when derived from the same tissue of origin, MSCs demonstrate prodigious donor-to-donor variation [42]. This may be a factor of donor health influencing MSC availability and function. Moreover, MSCs has also been shown intrapopulation heterogeneity in the multilineage differentiation potential [40]. For example, in single-cell clones from human ADSCs, 81% of the clones differentiated into at least one of the lineages, and 52% of the clones differentiated into two or more of the lineages [44]. Therefore, ASCs are a type of multipotent adult stem cells and not solely a mixed population of unipotent progenitor cells. Culture expansion also influences heterogeneity. Minimal culture expansion aids in reducing selective pressures that may alter cellular composition and functions. However, large-scale expansion of cells introduces bias into the culture process that is difficult to predict and/or control. Therefore, it is conceivable that large-scale expansion may select for or against a particular subpopulation, thereby enhancing or reducing potency, respectively. From these reasons, donor-to-donor and intrapopulation heterogeneity and effects of large-scale expansion on cellular composition and function may also be a contributing factor in MSC-based therapy. In this our study, we researched the properties of canine LDSCs using several assays in vitro. These data suggested that LDSCs might have functions similar to ADSCs, but it should be evaluated if in vitro results correlate with observed clinical outcomes.

## 5. Conclusion

These are the first data on the properties of LDSCs with ADSCs in terms of not only morphology, cell surface markers, and differentiation and proliferation abilities but also intracellular protein expression, secreted proteins, and cytokines. Canine LDSCs had variability in cell shape, proliferation ability, and adipogenesis differentiation among individuals, but LDSCs showed superior proliferation ability compared with ADSCs. Small differences in cellular proteomes and secretomes were observed between LDSCs and ADSCs. Together these in vitro assay results suggest that LDSCs could be one of the resources of MSCs. Further in vivo studies are required to evaluate the effects of LDSC functions on regenerative medicine.

## Figures and Tables

**Table 1:** Canine donor information.

Group	Case	Age (month)	Sex	Body weight (kg)	Collection site	Size (cm)
ADSC	A	13	M	11.0	Falciform ligament	—
	B	14	M	11.4	Falciform ligament	—
	C	15	M	11.6	Falciform ligament	—
	D	14	M	10.7	Falciform ligament	—

LDSC	E	90	F	5.3	Left femoral region	5 × 4 × 4
	F	105	F	52.0	Right axillary fossa	11 × 7 × 5
	G	90	MC	4.9	Left trunk	3 × 4 × 4
	H	169	FS	8.9	Right trunk	6 × 5 × 6
	I	178	FS	8.3	Right axillary fossa	10 × 8 × 4

**Table 2:** Flow cytometric analysis of cell surface markers.

	P2		P3		P4		P5		P6	
	ADSC	LDSC	ADSC	LDSC	ADSC	LDSC	ADSC	LDSC	ADSC	LDSC
CD29	94.5 ± 1.3	95.0 ± 2.2	95.8 ± 1.7	93.2 ± 1.6	90.9 ± 1.5	95.6 ± 4.4	92.1 ± 1.4	98.6 ± 0.4^∗^	93.2 ± 1.9	98.4 ± 0.5^∗^
CD44	99.6 ± 0.4	99.6 ± 0.4	99.2 ± 0.4	99.6 ± 0.3	98.8 ± 0.1	99.6 ± 0.2	99.5 ± 0.4	99.8 ± 0.2	99.4 ± 0.3	99.6 ± 0.3
CD90	95.2 ± 1.8	94.7 ± 3.0	92.6 ± 2.2	97.3 ± 1.0^∗^	90.8 ± 1.1	95.2 ± 2.4^∗^	89.9 ± 4.3	96.6 ± 1.3	89.3 ± 0.5	95.6 ± 1.4^∗^
CD34	0.2 ± 0.1	0.2 ± 0.2	0.1 ± 0.1	0.2 ± 0.2	0.3 ± 0.2	0.1 ± 0.1	0.3 ± 0.1	0.3 ± 0.2	0.2 ± 0.2	0.1 ± 0.1
CD14	0.2 ± 0.2	0.1 ± 0.1	0.1 ± 0.1	0.1 ± 0.1	0.2 ± 0.2	0.1 ± 0.1	0.1 ± 0.1	0.1 ± 0.1	0.1 ± 0.1	0.1 ± 0.1
CD45	0.1 ± 0.1	0.1 ± 0.1	0.1 ± 0.1	0.1 ± 0.1	0.1 ± 0.1	0.1 ± 0.1	0.1 ± 0.1	0.1 ± 0.1	0.1 ± 0.1	0.1 ± 0.1

Data are expressed as the percentage of positive cells (mean ± standard deviation). ^∗^*P* < 0.05, vs. ADSC.

**Table 3:** Protein spots that show different expression levels between ADSCs and LDSCs.

	Higher expression level	Spot number
A	ADSC (P2)	1, 2, 3, 5, 6, 7, 8, 9, 10, 11, 12, 13, 15
	LDSC (P2)	4, 7, 14

B	ADSC (P5)	16, 17, 18, 19, 23
	LDSC (P5)	11, 20, 21, 22

C	ADSC (P2)	1, 3, 5, 11, 12, 21, 22, 24, 26, 27, 29, 30, 31, 32, 33, 34, 35, 36, 38, 41, 42, 43, 45, 46
	ADSC (P5)	6, 23, 25, 28, 37, 39, 40

D	LDSC (P2)	14, 23, 47, 49, 50, 53
	LDSC (P5)	11, 15, 48, 51, 52

The spot numbers refer to numbers shown in Figure 7.

**Table 4:** Up- and downregulated proteins detected by iTRAQ.

ID	Protein
Proteins with 0.5 times or less expression in LDSC compared with ADSC	
G1K2D5	Calcyphosin
E2RR96	WAS protein family member 2
J9P4N7	Nephroblastoma overexpressed

Proteins with 1.5 times or more expression in LDSC compared with ADSC	
F1PHY1	Collagen alpha-2(I) chain
J9P0L0	Collagen type III alpha 1 chain
F6Y3P9	Gelsolin
F1Q0J3	Caldesmon 1
J9P8M2	Fibronectin
C7C419	Serpin peptidase inhibitor, clade H (heat shock protein 47), member 1 (collagen binding protein 1)
J9P5F0	Complement factor D
Q29393	Decorin
F6UYJ9	Calreticulin
F1PEM7	Insulin-like growth factor binding protein 2
F6PME1	Galectin
F1PJ74	Apolipoprotein E
E2RNR0	Osteoglycin
E2QUG4	Periostin
F1Q4D9	Retinol-binding protein
F1Q140	Podocan
J9NS29	Cystatin
H9GW59	AE binding protein 1
J9P2L4	HtrA serine peptidase 1
E2RKQ6	Galectin 3 binding protein
E2RJE0	Cartilage oligomeric matrix protein
W0RY37	Dickkopf 3 homolog
F1PMK7	Matrix metallopeptidase 2
F6V9A6	Collagen type VI alpha 3 chain
F1PLV6	Fibulin-1
E2RL80	Proline and arginine-rich end leucine-rich repeat protein
E2R6Q7	Cathepsin B
F1PLK4	Angiopoietin-like 4
F1PCT2	Mannose receptor C-type 2
F6Y2H4	Serpin family E member 2
F1PYX9	Serpin family G member 1
K0J6C5	Beta-N-acetylhexosaminidase beta subunit mRNA (fragment)
E2RPB8	C-type lectin domain family 3 member B
A0A346JM01	Protein S100
F1PE64	Calsyntenin 1
E2R0R3	Semaphorin 3C
J9NWK3	Sushi, von Willebrand factor type A, EGF, and pentraxin domain-containing 1
E2RF76	Chordin like 1
F1P6E1	Complement C1s
F1P903	Complement C1r
E2R599	Carboxypeptidase Q
J9P127	Thymosin beta
E2QXR8	RB binding protein 4, chromatin remodeling factor
J9P309	Actin-related protein 2/3 complex subunit 3
E2QY46	Dicarbonyl and L-xylulose reductase
E2R612	EGF containing fibulin extracellular matrix protein 1
F1PHS6	Peptidase inhibitor 16
F1PFZ5	Milk fat globule-EGF factor 8 protein
E2RC23	Procollagen C-endopeptidase enhancer
F1PG65	LIM domain 7
F1PAR9	NPC intracellular cholesterol transporter 2
E2RT85	Collagen type XIV alpha 1 chain
J9NYC0	Microfibril-associated protein 4
A0A077LQA5	Tubulin alpha chain
E2RNR9	Osteomodulin
E2RNB6	Crystallin alpha B
G1K2A7	Cathepsin K
A1DZY5	Diablo IAP-binding mitochondrial protein
F1Q0H0	N-Acylglucosamine 2-epimerase
E2QXA5	Thymosin beta
F1PZ83	Prostaglandin I2 synthase
J9NXV3	Vitrin
J9P1S2	Ras converting CAAX endopeptidase 1
F1PHF0	N-Acetylgalactosamine-6-sulfatase
E2RQE3	Proline-rich coiled-coil 2A
E2QX06	Transketolase-like 1
J9NXV9	BCAR1, Cas family scaffold protein
J9P2J8	Caldesmon 1
F1P6H7	Fibronectin
F1PDX9	T-complex protein 1 subunit gamma
F1PYE3	Canine mammary tumor
F1PYM4	Insulin-like growth factor binding protein acid labile subunit
F1PKX2	ABI family member 3 binding protein
E2RJW9	Adducin 1
E2QZE7	Adducin 3
D3YJ60	Chitinase 3-like 1
E2RJ75	WNT1 inducible signaling pathway protein 2
E2R8E3	Uncharacterized protein
F1PAH7	Eukaryotic translation initiation factor 4 gamma 2
F1PQM7	Tetraspanin
F1PYN7	Tumor protein p73
F1PHJ0	Solute carrier family 30 member 9
Proteins with 0.5 times or less expression in ADSC compared with LDSC	
F6Y3P9	Gelsolin
J9P5F0	Complement factor D
E2RNR0	Osteoglycin
A0A346JM01	Protein S100
J9P127	Thymosin beta
E2R612	EGF containing fibulin extracellular matrix protein 1
E2RNR9	Osteomodulin
F1Q0H0	N-Acylglucosamine 2-epimerase
F1PYN7	Tumor protein p73

Proteins with 1.5 times or more expression in ADSC compared with LDSC	
E2QW13	Inhibin subunit beta A
E2RAN6	Fructose-bisphosphatase 1
F1PKN7	Acetylserotonin O-methyltransferase like
J9P1A8	Phospholipase A2 activating protein
F1PF82	Spermine synthase O
E2RBV8	Heterogeneous nuclear ribonucleoprotein A0
E2RGF3	Na(+)/H(+) exchange regulatory cofactor NHE-RF
E2RIL1	Spectrin repeat containing nuclear envelope family member 3
E2RGR3	Stromal antigen 2
E2R0Y4	Cleavage and polyadenylation specific factor 7
F1PWP8	Uncharacterized protein
F1PQ43	Acidic leucine-rich nuclear phosphoprotein 32 family member A
F6XRK3	Uncharacterized protein
F1PP26	Family with sequence similarity 120A
E2R3N2	Delta-1-pyrroline-5-carboxylate synthase
J9NWK4	CDC42 effector protein 5
F2Z4P2	Ribosomal protein L7a
Q867A1	Laminin alpha 3 (fragment)
F6Y4X7	Metaxin 1
J9JHN4	Uncharacterized protein
F1PB05	Tsukushi, small leucine-rich proteoglycan

**Table 5:** Comparison of cytokine levels in conditioned medium between ADSCs and LDSCs.

	ADSC	LDSC
IL-2	0.6 (0-1.4)	0.6 (0-1.3)
IL-6	1.4 (1.2-1.5)	1.3 (1.2-1.4)
IL-8	38.2 (24.3-50.2)	29.3 (18.1-43.1)
IL-10	12.3 (1.3-35.1)	10.5 (4.3-28.3)
GM-CSF	0 (not detected)	0 (not detected)
MCP-1	167.7 (51.2-402.9)	399.7 (226.0-470.9)
RAGE	1.2 (0-3.2)	0.6 (0-2.2)
SCF	3.1 (0-3.8)	2.2 (0-3.1)
TNF*α*	0.5 (0-2.2)	0.3 (0-1.7)
VEGF-A	549.7 (253.4-1049.1)	508.3 (35.2-731.3)
EPO	1118 (684-1435)	946 (785-1272)
FGF-7	18.1 (0-32.2)	24.5 (0-38.2)
HGF	85.3 (19.5-109.4)	108.6 (36.6-143.2)
HGF R	103.2 (60.7-145.1)	74.9 (50.2-106.3)
IFN*γ*	54.4 (40.4-71.0)	54.5 (27.4-82.6)
IL-1*β*	14.2 (12.7-20.2)	17.8 (9.8-23.0)
IL-12p40	25.5 (4.7-45.3)	20.8 (2.8-40.4)
IL-17A	23.7 (18.7-30.8)	24.8 (15.3-34.8)
MIP-1*β*	77.8 (64.2-104.2)	74.0 (66.1-98.2)
TNF RI	21.6 (0-99.7)	19.0 (5.6-48.6)

Data are shown as the median and range. GM-CSF was not detected in conditioned medium of ADSCs and LDSCs. Concentrations of 19 other cytokines showed no differences between LDSC-CM and ADSC-CM.

## Data Availability

The data used to support the findings of this study are included within the supplementary information files.

## Supplementary Materials

Supplementary Materials Supplementary Material 1: proteins detected in conditioned medium of ADSCs and LDSCs by iTRAQ. Supplementary Material 2: Gene Ontology analysis of detection proteins in ADSC- and LDSC-CM.
